# MicroRNA-147a Targets SLC40A1 to Induce Ferroptosis in Human Glioblastoma

**DOI:** 10.1155/2022/2843990

**Published:** 2022-07-30

**Authors:** Peng Xu, Fei-Hang Ge, Wen-Xin Li, Zhen Xu, Xue-Li Wang, Jing-Lan Shen, Ai-Bo Xu, Rong-Rong Hao

**Affiliations:** ^1^The Third Affiliated Hospital of Zhejiang Chinese Medical University, Hangzhou, 310000 Zhejiang, China; ^2^Hangzhou Chinese Academy of Sciences-Hangzhou Medical College Advanced Medical Technology Institute, Hangzhou, 310018 Zhejiang, China; ^3^Dongyang people's Hospital, Jinhua, 322103 Zhejiang, China; ^4^College of Pharmaceutical Science, Zhejiang University of Technology, Hangzhou, 310032 Zhejiang, China; ^5^Hangzhou Medical College, Hangzhou, 310014 Zhejiang, China

## Abstract

**Objective:**

Glioblastoma is one of the most common malignant tumors in the brain, and these glioblastoma patients have very poor prognosis. Ferroptosis is involved in the progression of various tumors, including the glioblastoma. This study aims to determine the involvement of microRNA (miR)-147a in regulating ferroptosis of glioblastoma in vitro.

**Methods:**

Human glioblastoma cell lines were transfected with the inhibitor, mimic and matched negative controls of miR-147a in the presence or absence of ferroptotic inducers. To knock down the endogenous solute carrier family 40 member 1 (SLC40A1), cells were transfected with the small interfering RNA against SLC40A1. In addition, cells with or without the miR-147a mimic treatment were also incubated with temozolomide (TMZ) to investigate whether miR-147a overexpression could sensitize human glioblastoma cells to TMZ chemotherapy in vitro.

**Results:**

We found that miR-147a level was decreased in human glioblastoma tissues and cell lines and that the miR-147a mimic significantly suppressed the growth of glioblastoma cells in vitro. In addition, miR-147a expression was elevated in human glioblastoma cells upon erastin or RSL3 stimulation. Treatment with the miR-147a mimic significantly induced ferroptosis of glioblastoma cells, and the ferroptotic inhibitors could block the miR-147a mimic-mediated tumor suppression in vitro. Conversely, the miR-147a inhibitor prevented erastin- or RSL3-induced ferroptosis and increased the viability of glioblastoma cells in vitro. Mechanistically, we determined that miR-147a directly bound to the 3′-untranslated region of SLC40A1 and inhibited SLC40A1-mediated iron export, thereby facilitating iron overload, lipid peroxidation, and ferroptosis. Furthermore, miR-147a mimic-treated human glioblastoma cells exhibited higher sensitivity to TMZ chemotherapy than those treated with the mimic control in vitro.

**Conclusion:**

We for the first time determine that miR-147a targets SLC40A1 to induce ferroptosis in human glioblastoma in vitro.

## 1. Introduction

Glioblastoma is one of the most common malignant tumors in the brain and usually has poor prognosis, with a median survival time around 15 months even receiving standard treatment regimes (e.g., surgical resection, chemotherapy, and radiotherapy) [1–3]. Cell death plays critical roles in tissue homeostasis during development and disease progression. Ferroptosis is a novel, nonapoptotic form of programmed cell death, and mainly characterized by the iron-dependent accumulations of toxic lipid reactive oxygen species (ROS), which is morphologically, biochemically, and genetically distinct from other forms of cell death, such as apoptosis, necrosis, and autophagy [4–7]. Solute carrier family 40 member 1 (SLC40A1, also known as FPN1) is the only discovered mammalian iron efflux transporter in the plasma membrane and mediates iron releases from cells. SLC40A1 deficiency results in intracellular iron overload and lipid peroxidation, thereby inducing ferroptotic cell death [8, 9]. Recent studies have identified an essential role of ferroptosis in the progression of various human tumors, including the glioblastoma [10–12]. In addition, expressions of ferroptosis-related genes correlate with the occurrence and development of glioblastoma and can predict the prognosis and provide personalized treatment schedules for clinical patients [13]. Zhang et al. demonstrated that inducing ferroptosis significantly inhibited tumor growth, and moreover, igniting ferroptosis also prevented temozolomide (TMZ) chemoresistance of glioblastoma cells [14, 15]. These findings reveal that targeting ferroptosis may help to develop novel therapeutic approaches to treat glioblastoma.

microRNAs (miRNAs) are kinds of endogenous gene regulators through binding to the 3′-untranslated region (UTR) of targeted messenger RNAs, and play critical roles in a broad range of biological processes, including ferroptosis and glioblastoma [16–19]. Wang et al. previously revealed that miR-210-3p level was elevated in human glioblastoma tissues and positively correlated with the clinic stage. And manipulating miR-210-3p could regulate the apoptotic rate and progression of human glioblastoma cells in vitro [20]. In addition, Bier et al. demonstrated that miR-504 was significantly downregulated in human glioma stem cells and glioblastoma tissues, and that miR-504 overexpression in glioma stem cells effectively inhibited their self-renewal, migration, and the expression of mesenchymal markers, thereby preventing the progression of glioblastoma in vitro [21]. And findings from Zhang et al. also showed that t miR-1193 inhibition could sensitize human glioblastoma cells with DNA-PKcs deficiency in vitro [22]. miR-147a is implicated in many human tumors, including epithelial ovarian cancer and nonsmall cell lung cancer [23, 24]. However, the role of miR-147a in human glioblastoma remains unclear. The present study aims to investigate the involvement of miR-147a in regulating ferroptosis of human glioblastoma cells in vitro.

## 2. Materials and Methods

### 2.1. Chemicals and Antibodies

The micrON hsa-miR-147a mimic (#miR10000251-1-5), the mimic control (MCtrl, #miR1N0000001-1-5), the micrOFF hsa-miR-147a inhibitor (#miR20000251-1-5), and the inhibitor control (ICtrl, #miR2N0000001-1-5) were synthetized by Guangzhou RiboBio Co., Ltd. (Guangzhou, China). Lipofectamine™ 3000 transfection reagent (#L3000015) and 6-carboxy-2',7'-dichlorodihydrofluorescein diacetate (DCFH-DA, #C2938) were purchased from Thermo Fisher Scientific (Waltham, MA, USA). Erastin (#S7242), RSL3 (#S8155), ferrostain-1 (Fer-1, #S7243), and liproxstatin-1 (Lip-1, #S7699) were purchased from Selleck Chemicals (Houston, TX, USA). Dimethyl sulfoxide (DMSO, #D2650), temozolomide (TMZ, #T2577), cell counting kit-8 (CCK-8, #96992), and lipid peroxidation assay kit (#MAK085) were purchased from Sigma-Aldrich (St. Louis, MO, USA). Human 5-hydroxyeicosatetraenoic acid (5-HETE) ELISA kit (#CSB-E17006h) was purchased from CUSABIO (Houston, TX, USA). Lactate dehydrogenase (LDH) assay kit (#ab65393), colorimetric iron assay kit (#ab83366), 12-HETE ELISA Kit (#133034), 15-HETE ELISA Kit (#133035), and glutathione (GSH) detection assay kit (#ab112132) were purchased from Abcam (Cambridge, UK). Adenosine 5'-triphosphate (ATP) detection assay kit (#S0026) was purchased from Beyotime (Shanghai, China). The adenovirus carrying either full-length human SLC40A1 (AdSLC40A1) or scramble control (AdCtrl) was generated by Shanghai GeneChem Co., Ltd. (Shanghai, China). Anti-SLC40A1 (#ab58695), antiferritin heavy polypeptide 1 (FTH1, #ab75972), antitransferrin receptor (TFR, #ab84036), and anti-*β*-actin (#ab8226) were purchased from Abcam.

### 2.2. Cell Culture and Treatment

Human glioblastoma cells, U87MG and A172, and human normal brain astroglia cells, HA1800 and SVGp12, were purchased from ATCC (Manassas, VA, USA), and maintained in Dulbecco's Modified Eagle Medium (DMEM) supplemented with 10% fetal bovine serum (FBS) [25, 26]. To overexpress miR-147a, cells were transfected with the miR-147a mimic (50 nmol/L) using Lipofectamine™ 3000 Transfection Reagent for 4 h according to the manufacturer's instructions and then maintained in fresh DMEM containing 10% FBS for an additional 72 h. To induce ferroptosis, cells were stimulated with erastin (5 *μ*mol/L) or RSL3 (2 *μ*mol/L) for 72 h, and DMSO was used as a negative control [27]. In addition, the miR-147a mimic-transfected U87MG or A712 cells were treated with Fer-1 (1 *μ*mol/L) or Lip-1 (0.2 *μ*mol/L) for 72 h to inhibit ferroptosis as previously described [27]. To investigate whether the miR-147a inhibitor could prevent ferroptosis, cells were transfected with the miR-147a inhibitor (50 nmol/L) for 4 h and then stimulated with erastin (5 *μ*mol/L) or RSL3 (2 *μ*mol/L) for an additional 72 h. To overexpress SLC40A1, cells were infected with AdSLC40A1 at a multiplicity of infection of 20 for 4 h and maintained in fresh DMEM containing 10% FBS for an additional 24 h before the miR-147a mimic transfection. In a separated study, cells with or without the miR-147a mimic transfection were treated with TMZ (100 *μ*mol/L) for 48 h to determine whether overexpressing miR-147a could sensitize glioblastoma cells to TMZ chemotherapy [28].

### 2.3. Measurements of Cell Viability and LDH Releases

Cell viability was measured using the CCK-8 at 450 nm as previously described [29–31]. LDH levels in the medium were detected to evaluate cell death according to the manufacturer's instruction [32, 33]. Briefly, the medium was collected and centrifuged at 600*g* to remove any insoluble materials, and then incubated with WST substrate mix and LDH reaction mix for 30 min at room temperature, with the absorbance detected at 450 nm.

### 2.4. Colony Formation Assay

For the measurement of colony formation, cells were cultured for 2 weeks, and then, the colonies were fixed by methanol for 30 min at room temperature, stained with 0.5% crystal violet for 15 min, and rinsed with tap water carefully [34–36]. The colonies were imaged and counted in a blinded manner.

### 2.5. Quantitative Real-Time PCR Analysis

Total RNA was isolated with TRIzol reagent, and the concentrations were measured using Nanodrop 2000c [37–40]. Next, 2 *μ*g total RNA was reversely transcribed to cDNA with a Maxima First Strand cDNA Synthesis Kit (Roche), and quantitative real-time PCR was performed using SYBR Green. All gene expression was normalized to *β*-actin or U6.

### 2.6. Western Blot

Total proteins were extracted with RIPA lysis buffer according to standard protocols and then subjected to 10% SDS-PAGE assay [41–43]. Next, the proteins were transferred onto PVDF membranes and incubated with indicating primary antibodies overnight at 4°C. The combination of horseradish peroxidase-conjugated goat secondary antibodies with electrochemiluminescence substrate was used to visualize the protein bands, and the data were analyzed by the Image Lab software (Version 6.0).

### 2.7. Detection of Oxidative Stress

Cellular ROS level was detected by a DCFH-DA probe as previously described [44–46]. Briefly, cell lysates were prepared and incubated with DCFH-DA (50 *μ*mol/L) for 30 min at 37°C, and then, the fluorescence was measured at an excitation/emission wavelength of 488/525 nm. To measure intracellular malondialdehyde (MDA), cells were lysed and centrifuged to obtain the cell-free supernatants, which were then incubated with TBA solution and detected at 532 nm according to the manufacturer's instructions.

### 2.8. Iron assay

A colorimetric iron assay kit was used to detect intracellular iron level according to the manufacturer's instructions. Briefly, cells were lysed and centrifuged, with the supernatants incubated with iron assay buffer for 30 min at 37°C and iron probe for an additional 1 h. The absorbance was measured at 593 nm and calculated as the iron level.

### 2.9. Measurements of 5-HETE, 12-HETE, and 15-HETE

The levels of 5-HETE, 12-HETE, and 15-HETE in the medium were determined using commercial kits according to the manufacturer's instructions.

### 2.10. GSH and GPX4 Activity Assays

Intracellular GSH level was measured using a commercial kit. Briefly, cells were incubated with thiol green dye for 20 min at 37°C, and then, the fluorescence intensity was detected at an excitation/emission wavelength of 490/525 nm. GPX4 activity was detected using phosphatidylcholine hydroperoxide as a substrate according to a recent study [27].

### 2.11. Measurements of Mitochondrial Content and ATP Level

Mitochondrial content was evaluated by measuring mitochondrial genomic DNA (mtDNA) level according to a previous study [29]. Briefly, a commercial DNeasy kit was used to extract genomic DNA from glioblastoma cells, and then, mtDNA level was measured by normalizing Ct values of MT-CO2 gene (encoded by mtDNA). In addition, mtDNA integrity was determined by the ratio of long to short fragments. Intracellular ATP level was measured by a commercial ATP assay kit according to the manufacturer's instructions.

### 2.12. Luciferase Reporter Assay

Wild-type or truncated human SLC40A1 3′-UTR were synthesized and subcloned into the pGL3-Basic plasmid, which were then cotransfected into HEK293T cells with the miR-147a mimic for 48 h [8, 38]. The luciferase activities were measured with a dual-luciferase reporter assay system (Promega) and normalized to Renilla luciferase.

### 2.13. Statistical Analysis

Data were expressed as the mean ± SD and analyzed by the SPSS software (Version 22.0). The means between 2 groups were compared using unpaired Student's *t*-tests, while one-way ANOVA followed by Tukey post-hoc analysis was performed for comparison among 3 or more groups. *P* < 0.05 was considered statistically significant.

## 3. Results

### 3.1. The miR-147a Mimic Suppresses Cell Survival and Induces Cell Death of Human Glioblastoma Cells

**Figure 1:**
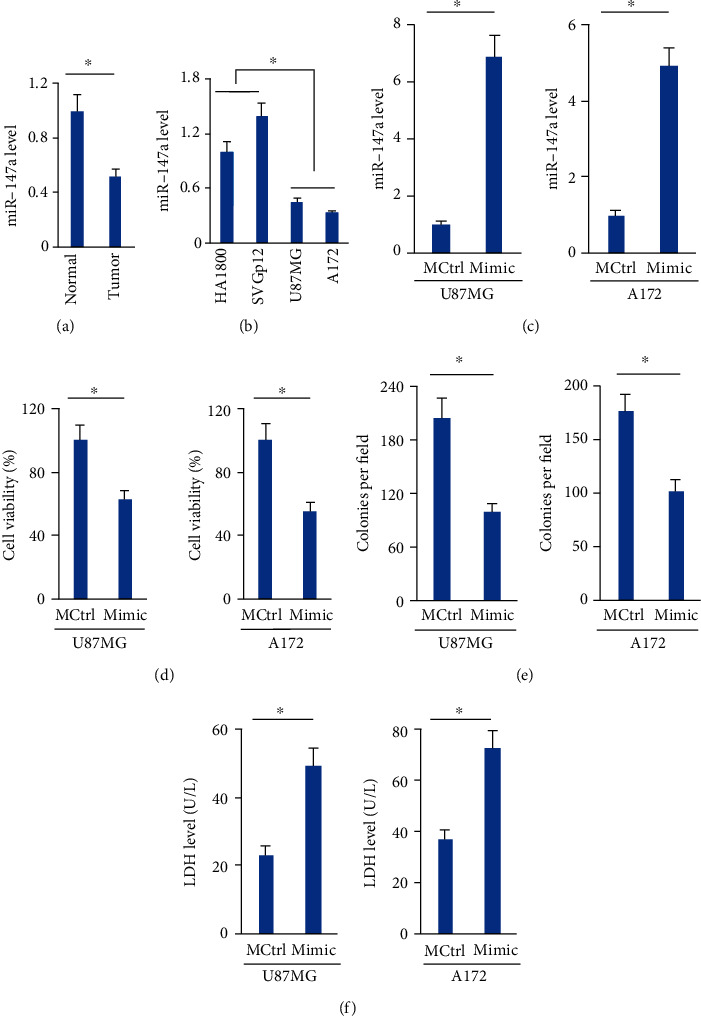
The miR-147a mimic suppresses cell survival and induces cell death of human glioblastoma cells. (a) miR-147a level in human glioblastoma tissues and normal brain tissues. (b) miR-147a level in human glioblastoma cells and normal brain astroglia cells. (c) miR-147a level in human glioblastoma cells treated with or without the miR-147a mimic. (d) Cell viability detected by CCK-8 assay. (e) Numbers of colonies. (f) LDH level in the medium from cells treated with or without the miR-147a mimic. *N* = 6 for each group. Data were expressed as the mean ± SD, and *P* < 0.05 was considered statistically significant.

To understand the role of miR-147a during the progression of human glioblastoma, we evaluated miR-147a expression in human glioblastoma tissues and cells. As shown in Figure 1(a), miR-147a level was significantly decreased in human glioblastoma tissue compared with normal brain tissue. And U87MG and A172 cells, two human glioblastoma cells, also displayed lower miR-147a expression (Figure 1(b)). Next, these two cells were treated with the miR-147a mimic to increase intracellular miR-147a expression (Figure 1(c)). Interestingly, the miR-147a mimic significantly suppressed the survival of U87MG and A172 cells, and cell growth was also inhibited, as determined by the decreased colony formation (Figures 1(d) and 1(e)). In addition, treatment with the miR-147a mimic also elevated LDH levels in the medium, indicating an increased cell death (Figure 1(f)). These results suggest that the miR-147a mimic suppresses cell survival and induces cell death of human glioblastoma cells.

### 3.2. The miR-147a Mimic Triggers Ferroptosis of Human Glioblastoma Cells

**Figure 2:**
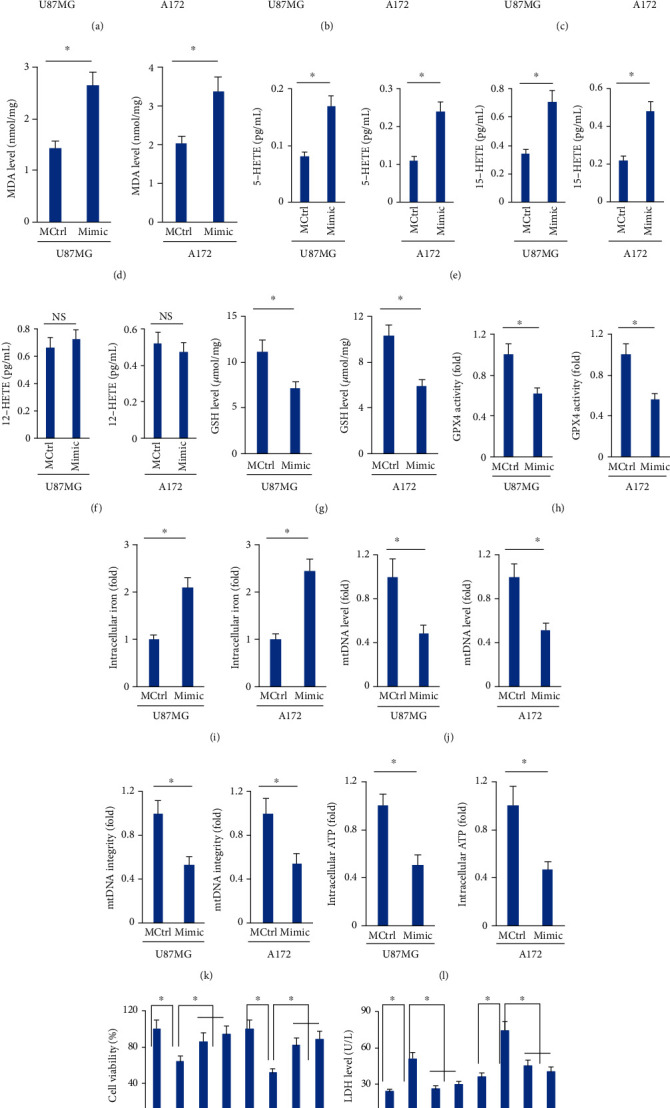
The miR-147a mimic triggers ferroptosis of human glioblastoma cells. (a–b) miR-147a level in human glioblastoma cells treated with erastin or RSL3. (c) ROS level detected by DCFH-DA probe. (d) MDA level in cells treated with or without the miR-147a mimic. (e–f) 5-HETE, 12-HETE, and 15-HETE levels in the medium from cells treated with or without the miR-147a mimic. (g–h) GSH level and GPX4 activity in U87MG and A172 cells. (i) Relative level of intracellular iron. (j–k) Quantification of mtDNA content and integrity. (l) Quantification of intracellular ATP level. (m) Cell viability and LDH releases in the miR-147a mimic-treated human glioblastoma cells with Fer-1 or Lip-1 protection. *N* = 6 for each group. Data were expressed as the mean ± SD, and *P* < 0.05 was considered statistically significant.

Ferroptosis is a novel form of programmed cell death and has been implicated in the pathogenesis of various human tumors, including the glioblastoma [47, 48]. As shown in Figures 2(a) and 2(b), the expression of miR-147a was elevated in human glioblastoma cells treated with ferroptotic inducers, erastin or RSL3. Next, we evaluated whether ferroptosis was involved in the miR-147a mimic-mediated tumor suppression. Interestingly, treatment with the miR-147a mimic significantly increased intracellular ROS generation, accompanied by an enhanced lipid peroxidation (Figures 2(c) and 2(d)). During ferroptosis, polyunsaturated fatty acids can be oxidized into HETEs [27]. Accordingly, we detected elevated releases of 5-HETE and 15-HETE to the medium in the miR-147a mimic-treated cells; however, no alteration of 12-HETE level was observed between groups (Figures 2(e) and 2(f)). In addition, the activities of GSH and downstream GPX4, two important molecules to scavenge excessive free radicals, were also suppressed by the miR-147a mimic (Figures 2(g) and 2(h)). Iron contributes to ROS generation and ferroptosis execution. As expected, U87MG or A172 cells treated with the miR-147a mimic displayed higher iron concentration compared to those treated with the MCtrl (Figure 2(i)). Mitochondria are major organelles involved in iron storage and utilization and are also implicated in the generation of intracellular ROS. As shown in Figures 2(j)–2(l), mitochondrial content and function are significantly compromised by the miR-147a mimic, as evidenced by the decreased mtDNA level, mtDNA integrity, and intracellular ATP level. To further investigate whether the miR-147a mimic-mediated tumor suppression was achieved through the induction of ferroptosis, the miR-147a mimic-transfected U87MG or A712 cells were incubated with Fer-1 or Lip-1 to inhibit ferroptosis. As shown in Figure 2(m), both Fer-1 and Lip-1 could abrogate the miR-147a mimic-induced tumor suppression, as evidenced by the increased cell viability and decreased LDH releases in U87MG and A712 cells. From the data, we conclude that the miR-147a mimic triggers ferroptosis of human glioblastoma cells.

### 3.3. The miR-147a Inhibitor Prevents Ferroptosis of Human Glioblastoma Cells

**Figure 3:**
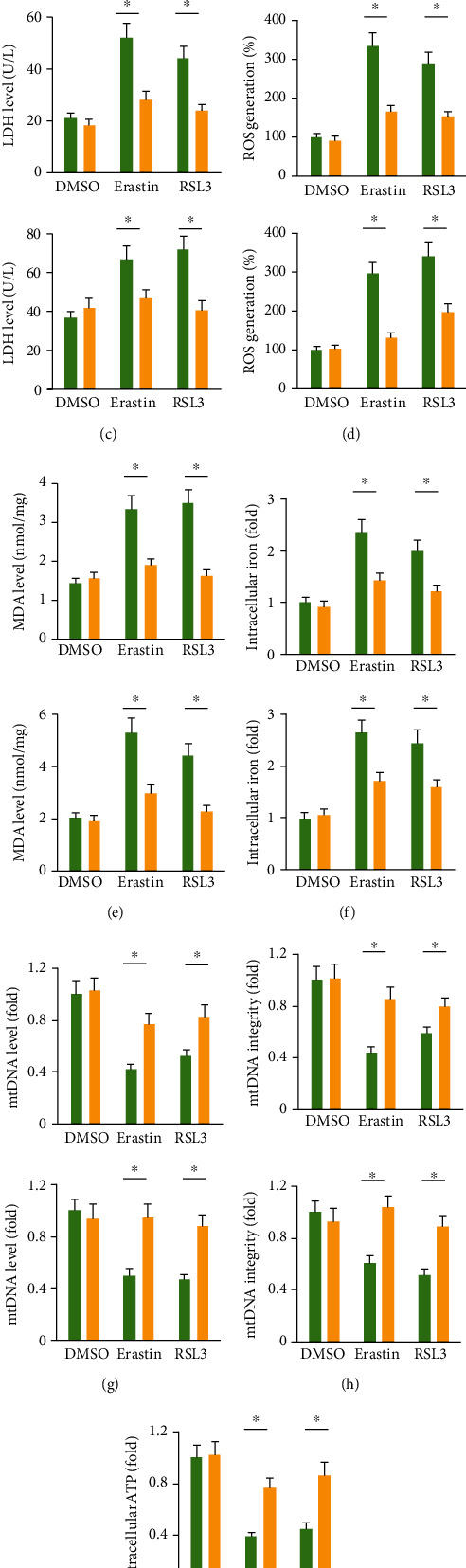
The miR-147a inhibitor prevents ferroptosis of human glioblastoma cells. (a) miR-147a level in human glioblastoma cells treated with or without the miR-147a inhibitor. (b) Cell viability detected by CCK-8 assay. (c) LDH level in the medium from cells treated with or without the miR-147a inhibitor. (d) ROS level detected by DCFH-DA probe. (e) MDA level in cells treated with or without the miR-147a inhibitor. (f) Relative level of intracellular iron. (g–h) Quantification of mtDNA content and integrity. (i) Quantification of intracellular ATP level. *N* = 6 for each group. Data were expressed as the mean ± SD, and *P* < 0.05 was considered statistically significant.

Then, we used the miR-147a inhibitor to suppress endogenous miR-147a expression (Figure 3(a)). As shown in Figures 3(b) and 3(c), treatment with the miR-147a inhibitor did not affect the survival of human glioblastoma cells under basal conditions, but prevented erastin- or RSL3-induced cell death, as evidenced by the increased cell viability and decreased LDH releases (Figures 3(b) and 3(c)). In addition, ROS generation and lipid peroxidation in erastin- or RSL3-treated U87MG or A172 cells were decreased by the miR-147a inhibitor (Figures 3(d) and 3(e)). Meanwhile, we found that erastin- or RSL3-induced increases of intracellular iron were also decreased in the miR-147a inhibitor-treated human glioblastoma cells (Figure 3(f)). In addition, the miR-147a inhibitor significantly prevented erastin- or RSL3-induced impairments of mitochondrial content and function (Figures 3(g)–3(i)). Our findings reveal that the miR-147a inhibitor prevents ferroptosis of human glioblastoma cells.

### 3.4. The miR-147a Mimic Elicits Ferroptosis of Human Glioblastoma Cells through Targeting SLC40A1

**Figure 4:**
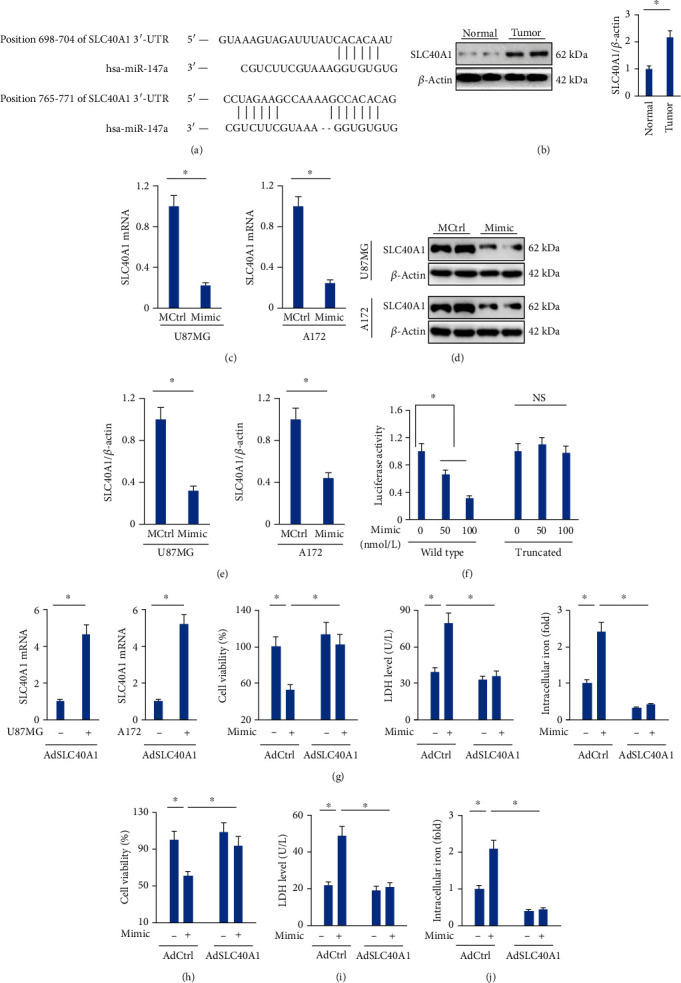
The miR-147a mimic elicits ferroptosis of human glioblastoma cells through targeting SLC40A1. (a) Sequence alignment of miR-147a and the 3′-UTR of SLC40A1. (b) SLC40A1 protein level in human glioblastoma tissues and normal brain tissues. (c–e) The mRNA and protein levels of SLC40A1 in cells treated with or without the miR-147a mimic. (f) Relative luciferase activity. (g) The mRNA level SLC40A1 in cells infected with or without AdSCL40A1. (h) Cell viability detected by CCK-8 assay. (i) LDH level in the medium from the miR-147a mimic-treated cells with or without SLC40A1 overexpression. (j) Relative level of intracellular iron. *N* = 6 for each group. Data were expressed as the mean ± SD, and *P* < 0.05 was considered statistically significant. NS indicates no significance.

Next, we tried to decipher the underlying mechanism that mediated the tumor-suppressive effects of the miR-147a mimic. As shown in Figure 4(a), using TargetScan software, we identify two putative binding sites of miR-147a in the 3′-UTR of SLC40A1, the only discovered mammalian iron efflux transporter in the plasma membrane [9]. And a significant increase of SLC40A1 expression was observed in human glioblastoma tissues (Figure 4(b)). Of note, treatment with the miR-147a mimic dramatically decreased the mRNA and protein levels of SLC40A in U87MG or A172 cells (Figures 4(c)–4(e)). Results from the luciferase reporter assay demonstrated that miR-147a directly bound to the 3′-UTR of SLC40A1 (Figure 4(f)). To further validate the necessity of SLC40A1, cells were preinfected with AdSLC40A1 to overexpress SLC40A1 and then transfected with or without the miR-147a mimic (Figure 4(g)). As shown in Figures 4(h) and 4(i), the decreased cell viability and increased LDH release in the miR-147a mimic-treated human glioblastoma cells are completely blocked by SLC40A1 overexpression (Figures 4(h) and 4(i)). In addition, the miR-147a mimic lost its role to induce iron accumulation in SLC40A1-overexpressed U87MG or A172 cells (Figure 4(j)). Intracellular iron homeostasis also depends on iron intake and storage, and we thus detected the expressions of FTH1 and TFR, two key proteins for iron storage and intake, respectively. As shown in Figure S1, the miR-147a mimic did not affect FTH1 or TFR protein levels. Taken together, we demonstrate that the miR-147a mimic elicits ferroptosis of human glioblastoma cells through targeting SLC40A1.

### 3.5. The miR-147a Mimic Sensitizes Human Glioblastoma Cells to TMZ Therapy

**Figure 5:**
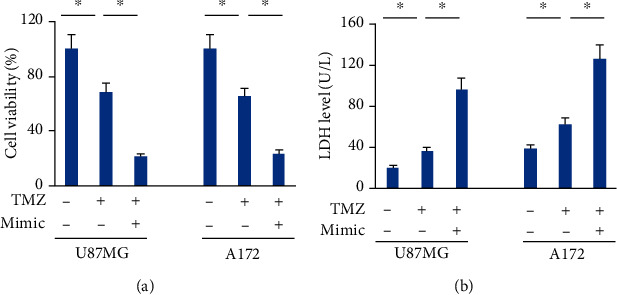
The miR-147a mimic sensitizes human glioblastoma cells to TMZ therapy. (a) Cell viability detected by CCK-8 assay. (b) LDH level in the medium from the miR-147a mimic-treated cells with or without TMZ stimulation. *N* = 6 for each group. Data were expressed as the mean ± SD, and *P* < 0.05 was considered statistically significant.

Given the fact that the miR-147a mimic could induce ferroptosis of U87MG or A172 cells, we finally determined whether the miR-147a mimic could sensitize human glioblastoma cells to TMZ therapy, a standard regime to treat human glioblastoma. Interestingly, the miR-147a mimic-treated cells displayed lower survival rate and more LDH releases upon TMZ stimulation, implying that the miR-147a mimic sensitizes human glioblastoma cells to TMZ therapy (Figures 5(a) and 5(b)).

## 4. Discussion

Cell death plays critical roles in the pathogenesis of tumor progression and chemoresistance. Ferroptosis is a novel form of programmed cell death and serves as an important target to treat various human tumors, including the glioblastoma [28]. In the present study, we found that miR-147a expression was decreased in human glioblastoma tissues and cells and that miR-147a overexpression by the miR-147a mimic could significantly inhibit survival and induce death in U87MG or A172 cells in vitro. In addition, we identified that miR-147a directly bound to the 3′-UTR of SLC40A1, induced intracellular iron accumulation, and ultimately resulted in lipid peroxidation and ferroptosis, and that SLC40A1 overexpression significantly blocked the miR-147a mimic-mediated tumor suppression in human glioblastoma cells in vitro. Moreover, we revealed that treatment with the miR-147a mimic could sensitize human glioblastoma cells to TMZ therapy in vitro. These findings define miR-147a as a promising therapeutic target to treat human glioblastoma.

Iron is well-accepted as an essential nutrient for the growth of mammalian cells, including multiple human tumor cells, and emerging epidemiological findings have demonstrated that excessive iron contributes to increased incidence, risk, and metastasis for human tumors [49]. The characteristic of iron dependence for tumor cells identify iron metabolism as a novel therapeutic target to treat human tumors. Ferroptosis is a newly discovered type of programmed cell death and is mainly induced by the excessive generation of toxic lipid ROS that depends on intracellular iron accumulation. Intracellular ferrous iron provokes the generation of cytotoxic lipid radicals and subsequently induces death of tumor cells. The regulation of intracellular iron homeostasis is finely orchestrated by a complex mechanism. Extracellular iron is carried by transferrin and then enters into cells by binding to TFR at cell surface, which is internalized by receptor-mediated endocytosis, trafficked in an endosome and stored in ferritin [50]. The export of intracellular iron is mainly achieved by SLC40A1, the only discovered mammalian iron efflux transporter in the plasma membrane [9, 51]. SLC40A1 expression is well-controlled by the small peptide hepcidin, and SLC40A1 deficiency causes the accumulation of intracellular iron [52]. Various studies have demonstrated that inhibiting SLC40A1 significantly prevented tumor cell growth by inducing ferroptosis. Tang et al. recently revealed that ubiquitin-specific protease 35 silence inhibited cell growth, colony formation, and tumor progression in lung cancer cells through inducing SLC40A1 degradation and ferroptotic cell death [27]. Accordingly, we herein demonstrated that miR-147a directly bound to the 3′-UTR of SLC40A1 and suppressed its expression, thereby promoting intracellular iron accumulation, lipid peroxidation, and ferroptosis of human glioblastoma cells in vitro.

miRNAs play versatile roles in various biological processes, including cell death and tumor progression [53, 54]. Guan et al. previously showed that miR-196 was upregulated in human glioblastoma tissues, and had prognostic significance for glioblastoma, rather than anaplastic astrocytomas and normal brains. Moreover, glioblastoma patients with higher miR-196 levels exhibited poorer survival, and an independent predictive role of miR-196 for overall survival in glioblastoma patients was also determined [55]. Wang et al. revealed that miR-25-3p overexpression facilitated the proliferation and TMZ resistance of glioblastoma cells and that exosomal miR-25-3p might also be a potential prognostic biomarker for glioblastoma patients [56]. Emerging studies have revealed the complex interaction between iron and noncoding RNAs, especially miRNAs. Ferritin keeps intracellular iron in a bioavailable and nontoxic form and contributes to intracellular iron equilibrium. Biamonte et al. and Sanzo et al. demonstrated that knocking down the FTH1 modulated the expression of a specific set of miRNAs [57, 58]. In addition, Sanzo et al. also identified some putative binding sites in 3′-UTR of FTH1 and ferritin heavy polypeptide 1 pseudogene 3, another member of the FTH1 gene family [59]. miR-147a is implicated in the pathogenesis of different human tumors. Wang et al. previously determined that miR-147a was induced by hypoxia through hypoxia-inducible factor-1*α* (HIF-1*α*), and then stabilized and accumulated HIF-1*α* protein to suppress tumor growth [60]. In addition, miR-147a overexpression could also reduce proliferation and promote apoptosis of nonsmall cell lung cancer cells [24]. Moreover, miR-147a inhibition significantly promoted the malignant phenotypes of cancers [61]. Yet, the role and mechanism of miR-147a in human glioblastoma and ferroptosis have not been well-elucidated. In the present study, we detected a significant reduction of miR-147a in human glioblastoma tissues and cells, while its expression was dramatically elevated by the ferroptotic stimulation. And the miR-147a mimic induced, while the miR-147a inhibitor prevented ferroptosis, thereby regulating the growth of U87MG and A172 cells in vitro. More importantly, we demonstrated that pretreatment with the miR-147a mimic could sensitize human glioblastoma cells to TMZ chemotherapy in vitro, indicating that the miR-147a mimic could be used as adjuvant agent during TMZ chemotherapy in clinic.

In summary, our data for the first time determine that miR-147a targets SLC40A1 to induce ferroptosis in human glioblastoma in vitro.

## Data Availability

Data supporting the findings of this work are available from the corresponding author upon reasonable request.

## Supplementary Materials

Supplementary Materials Figure S1. The miR-147a mimic does not affect iron intake and storage of human glioblastoma cells.
