# Supplementary material for: MicroRNA-147a Targets SLC40A1 to Induce Ferroptosis in Human Glioblastoma
Source: Anal Cell Pathol (Amst). 2022 Jul 30;2022:2843990. doi: 10.1155/2022/2843990 (PMC9356897; doi:10.1155/2022/2843990)
Supplement: Supplementary Materials — Figure S1. The miR-147a mimic does not affect iron intake and storage of human glioblastoma cells. [file 2843990.f1.docx]

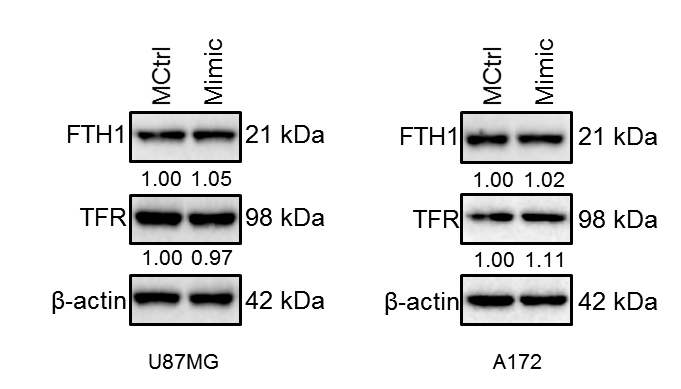


**Figure S1. The miR-147a mimic does not affect iron intake and storage of human glioblastoma cells.** Protein levels of FTH1 and TFR in the miR-147a mimic-treated human glioblastoma cells. *N* = 6 for each group. Data were expressed as the mean ± S.D., and *P* < 0.05 was considered statistically significant.
